# Treatment of Typhus

**Published:** 1818-02

**Authors:** R. Moulson

**Affiliations:** Physician to the Halifax General Dispensary


*Treatment of Typhus;* by R. MOULSON, M. D. Physician to the Halifax General Dispensary.

THE newspapers have recently been disseminating fears and tremblings amongst their readers respecting the Typhus fever here and there attacking families, filling Fever Houses, and diminishing the bulk of mankind by its ravages. Those whom it most generally attacks, deservedly merit a scourge of some kind for the filth and dirt that is courted by them; and next to them, the proprietors of the dwellings of the poor, for huddling stones together that are to form habitations for their fellow creatures, from which currents of fresh air are entirely excluded, and appropriate conveniences necessary to dwelling-houses are supplanted by the first hole before each door, making a receptacle for filth and stenches, so conducive to the undermining of constitutions, and to the dissemination of diseases when once formed. Exemption from fevers is most likely to be obtained by cleanliness and pure air. To mitigate disease by the most simple and effectual means deservedly merits our most serious consideration; and therefore, I shall be happy to add my mite to the treatment of typhus found to be most successful, should it be thought worthy of a place in your Journal.

The variety of forms in which Typhus makes its appearance, depends in a very great measure upon the local situation of the poor sufferers; and for that reason, no characteristic symptoms can be detailed as being universal. If the pulse be considered as a criterion as to whether bleeding be proper or not, I think that we shall very frequently be misled: and the symptoms which appear to demand more serious attention, are, the pains in the head, state of the bowels, and heat of skin. Whenever I have been called to a patient labouring under typhus, however great the prostration of strength, if the pain in the head be violent, I have ordered the temporal artery to be opened; and where that could not be conveniently done, eight or ten leeches have been applied to the head and allowed to bleed freely; for I consider the prostration of strength to be chiefly occasioned by an undue quantity of blood being determined to the vessels of the brain. If, on the following day, (which has seldom been the case) the pain has recurred, again has recourse been had to bleeding from the head; and this has been done in cases where general bleeding from the arm would very probably have proved injurious, if not fatal. Some cases in robust persons were bled from the arm, but their recovery was evidently retarded, and general debility accompanied their convalescence. From several experiments I made two years ago, I found that arteriotomy was much less debilitating than venæsection; since which time, I have directed an artery to be opened, in cases where I feared to open a vein. If the bowels were confined, I opened them with the following bolus. ℞. Hydrarg. submuriat. gr. iv. Jalapæ pulv. gr. xv. Mucil. g. acaciæ q. s. ut ft. bolus. On the contrary, if the bowels were open, I omitted the purgative bolus and commenced giving the adjoined pills and mixture.

℞. Hydrarg. submuriat. gr. iij. Pulv. antimonalis gr. j. Opii purificati gr. $\frac{1}{2}$. Confect. aromat. q. s. ut ft. pilulâ quartâ quâque horâ cum cochleariis duobus misturæ sequentis.

℞. Infusi rosæ oſs. Magnesiæ sulphat. ℥j. Magnesiæ ʒiſs. M. ft. mistura.

So soon as the mouth became affected, which frequently occurred on the third or fourth day of taking the medicines, the unpleasant symptoms, such as, black furred tongue, burning heat of skin, thirst, fœtid stools, &c. gradually vanished; and the only medicines afterwards required, were some of the vegetable bitter infusions to reconcile the stomach to receive more nutritious diet; the decoction of bark was sometimes given, but very seldom was the powder of bark necessary. Wine was out of the question, except to those who could afford it when in a state of convalescence. So long as the heat of skin remained pungent, sponging the body with cold water and vinegar was regularly attended to; and under this treatment all, as yet, have recovered. Blisters in some cases were applied to the head, but I cannot say that they afforded so much relief as cloths wrung out of cold water kept constantly applied there.

Before I had the gratification of perusing Dr. Armstrong's valuable work on Typhus, I believed that fevers depended upon the balance of the circulation being destroyed; and it is very astonishing to me, that Dr. Clutterbuck's Inquiry into the Seat and Nature of Fever, did not meet with more supporters when it came from the press; but it is easily to be accounted for, as many of those men who will not dissect for themselves, pay not the least attention to what is advanced contrary to the dogmas of the schools, for they consider any new light thrown upon a subject, as the flight of some fanciful imagination that conceives more than it brings forth. I am sorry to say, that in the circle of my own acquaintance I have seen this remark verified.

If Typhus (as is conceived by some) depends upon local inflammation of the brain, why are not the symptoms the same as in phrenitis? In the worst cases of Typhus, the patients appear inclined more to an apoplectic state than otherwise.

For my part, I conceive that inflammation of the brain occurs extremely seldom in Typhus, but that congestion of the vessels of the brain, on the other hand, is always present. I may be wrong, yet as I have acted under that idea, and been successful, I still retain that opinion. In those bodies that I have opened dying of spasmodic diseases, and where I have seen the vessels of the spine gorged with blood, I have never as yet seen any thing to make me look upon it as inflammation.*

For this digression I beg to apologize, but I should wish some of your ingenious Correspondents to decide, How long inflammation can exist in an internal part without destroying that part? and, can active inflammation exist beyond a certain number of days? say, nine. The term Chronic Inflammation I discard.

*December* 10, 1817.

---

* It may naturally be asked, what I conceive constitutes the difference between inflammation and congestion, if I admit having seen vessels gorged with blood, and yet say, inflammation was not present. The following experiment will explain my views of the subject much better than I can describe them upon paper. Bleed a horse to death from a *vein*, say the Jugular, and afterwards examine the blood-vessels of the mesentery, and compare them with the appearance of the veins of the intestines in a human subject dead of enteritis—the difference will be obvious.

